# Engineering Chimeric Antigen Receptor T-Cells for Racing in Solid Tumors: Don’t Forget the Fuel

**DOI:** 10.3389/fimmu.2017.00267

**Published:** 2017-04-03

**Authors:** Melita Irving, Romain Vuillefroy de Silly, Kirsten Scholten, Nahzli Dilek, George Coukos

**Affiliations:** ^1^The Ludwig Institute for Cancer Research, University of Lausanne, Epalinges, Switzerland; ^2^Swiss Institute of Bioinformatics, University of Lausanne, Lausanne, Switzerland; ^3^Department of Oncology, University Hospital of Lausanne (CHUV), Lausanne, Switzerland

**Keywords:** immunotherapy, chimeric antigen receptor, T-cells, gene-engineering, immunometabolism, solid tumors, tumor microenvironment

## Abstract

T-cells play a critical role in tumor immunity. Indeed, the presence of tumor-infiltrating lymphocytes is a predictor of favorable patient prognosis for many indications and is a requirement for responsiveness to immune checkpoint blockade therapy targeting programmed cell death 1. For tumors lacking immune infiltrate, or for which antigen processing and/or presentation has been downregulated, a promising immunotherapeutic approach is chimeric antigen receptor (CAR) T-cell therapy. CARs are hybrid receptors that link the tumor antigen specificity and affinity of an antibody-derived single-chain variable fragment with signaling endodomains associated with T-cell activation. CAR therapy targeting CD19 has yielded extraordinary clinical responses against some hematological tumors. Solid tumors, however, remain an important challenge to CAR T-cells due to issues of homing, tumor vasculature and stromal barriers, and a range of obstacles in the tumor bed. Protumoral immune infiltrate including T regulatory cells and myeloid-derived suppressor cells have been well characterized for their ability to upregulate inhibitory receptors and molecules that hinder effector T-cells. A critical role for metabolic barriers in the tumor microenvironment (TME) is emerging. High glucose consumption and competition for key amino acids by tumor cells can leave T-cells with insufficient energy and biosynthetic precursors to support activities such as cytokine secretion and lead to a phenotypic state of anergy or exhaustion. CAR T-cell expansion protocols that promote a less differentiated phenotype, combined with optimal receptor design and coengineering strategies, along with immunomodulatory therapies that also promote endogenous immunity, offer great promise in surmounting immunometabolic barriers in the TME and curing solid tumors.

## Natural Tumor Immunity and Response to Immunotherapy

### Immune Checkpoint Blockade

T lymphocytes play a critical role in tumor immunity through the recognition of tumor-associated antigens processed and presented as peptides at the cell surface by major histocompatibility complex (MHC) molecules (1). For various tumor types, the presence of tumor-infiltrating T lymphocytes (TILs) predicts longer disease-free survival and overall patient survival (2–6). Cancers employ numerous mechanisms of immune evasion (7) that dampen T-cell activity, which in some patients can be successfully reversed by monoclonal antibodies (mAbs) targeting immune checkpoints, such as cytotoxic T lymphocyte-associated protein 4 (CTLA-4), and the programmed cell death 1 (PD-1)/PD ligand 1 (PD-L1) axis. These immunomodulatory mAbs have enabled regression of a range of malignancies including melanoma (8, 9), lung (10), bladder (11), Hodgkin’s lymphoma (12), renal-cell carcinoma (13), ovarian (14), as well as gastrointestinal and endometrial cancers with DNA mismatch-repair defects (15), thus providing proof that the majority of solid tumor types can be spontaneously recognized by the host’s T-cells. PD-1 inhibition alone is active in about 30% of cancer patients, but in combination with CTLA-4 the fraction of responding metastatic melanoma patients increases to 57% (16). Notably, clinical responses to immunotherapy are usually associated with durability (17).

A number of studies have attempted to elucidate mechanisms underlying resistance to checkpoint blockade. The presence of CD8^+^ T-cells within the tumor or its invasive margin is a key requirement for responses to PD-1 inhibition (18). Many studies indicate that the neoantigen load may also be an influencing factor (19–22). However, as the case of Merkel-cell carcinoma (MCC) has shown, the quality of tumor epitopes and the corresponding TILs, and not only the mutation rate, can confer sensitivity to checkpoint blockade; MCC that is virally induced and having a low mutation rate responds similar to checkpoint blockade as MCC caused by ultraviolet radiation (23, 24). The presence of CD8^+^ TILs has been linked to the expression of a type I interferon (IFN) signature in tumors (25, 26) and the recruitment of a subset of CD103^+^/CD8α^+^ DCs driven by the transcription factor Batf3 (27, 28). WNT/β-catenin signaling upregulation may be one of the tumor cell-intrinsic pathways driving a non-T-cell inflamed phenotype, at least in metastatic melanoma (29) for which a genetically engineered mouse model revealed reduced expression of the chemokine CCL4, impaired recruitment of Batf3^+^ DCs and T-cells, and resistance to checkpoint blockade (29). Interestingly, commensal bacteria in the gut can also shape innate immunity and responses to immune checkpoint therapy (30–32). Finally, metabolic circuitries play an important role in regulating immune function in tumors. It has recently been demonstrated that CTLA-4, PD-1, and PD-L1 blockade can restore glucose levels in the tumor microenvironment (TME), thereby improving T-cell fitness. Anti-PD-L1 mAb was specifically shown to block the mechanistic target of rapamycin [mTOR, a central regulator of metabolism and physiology (33)] and decrease the expression of glycolytic enzymes in tumor cells (34). Further, for PD-L1^+^ renal-cell carcinoma patients, non-responsiveness to PD-1 blockade has been associated with metabolic gene upregulation as well as of solute transport functions such as UGT1A family members, whereas responders present an immune response profile including upregulation of CCL3, a chemokine involved in leukocyte migration (35).

### TIL and Chimeric Antigen Receptor (CAR) T-Cell Therapy

Another powerful form of immunotherapy is adoptive T-cell therapy (ACT), which entails the *ex vivo* expansion of tumor-specific T-cells and their infusion into a patient. For TIL therapy, in which T lymphocytes are enriched from tumor biopsies, patients are typically lymphodepleted and receive high-dose interleukin-2 (IL-2) (36–38). TIL therapy has proven successful in advanced metastatic melanoma, mediating objective responses in about 50% of patients, and durable complete responses in up to 20% of patients receiving a single TIL infusion (36). It is now evident that in the case of metastatic melanoma an important target of TILs are mutated gene products (39). TIL therapy has also been anecdotally successful in common carcinomas (40), suggesting that this approach could be applied to other solid tumor indications. For various reasons, however, ranging from tumor vasculature barriers to a lack of type I IFN signaling, not all tumors are infiltrated by T-cells at baseline (27, 41–43).

In the absence of endogenous T-cell infiltrate due to aberrant antigen processing and presentation, for example, which precludes the use of TIL therapy and immune checkpoint blockade, a promising solution for treating cold tumors is the transfer of mAb-modified T-cells, so-called CAR T-cells (39). In recent years, CD19-targeted CAR T-cell therapy has yielded spectacular clinical responses against hematologic liquid tumors (44), including up to 90% complete response in relapsed or treatment-refractory acute lymphoblastic leukemia (ALL) patients (45). In the solid TME, however, T-cells face a battery of physical and immunometabolic barriers (46, 47), to which CAR T-cells, like endogenous T-cells, are vulnerable (48, 49). CAR T-cells may thus similarly require combinatorial regimens of immunomodulation such as kinase inhibitors (50), chemotherapy (51), radiotherapy (RT) (52), or checkpoint blockade (53), to unleash their full therapeutic potential (54–56). CAR T-cells can also be armored through additional gene modification (57). For example, they have been coengineered to express stimulatory ligands, such as CD40 ligand (CD40L) (58), or to secrete stimulatory cytokines, such as IL-12 (57), for improved antitumor responses. With an emerging awareness of the role played by metabolism in both cancer progression and T-cell activity in the TME (59), it is apparent that further development of CAR T-cell therapy for maximizing functionality in harsh, nutrient-depleted conditions is critical. Here, we review the design and function of CAR T-cells, immunometabolic barriers in the solid TME, and different *ex vivo* expansion, coengineering and combinatorial therapy approaches for overcoming them.

## CAR T-Cell Engineering

### Basic CAR Design

**Figure 1:**
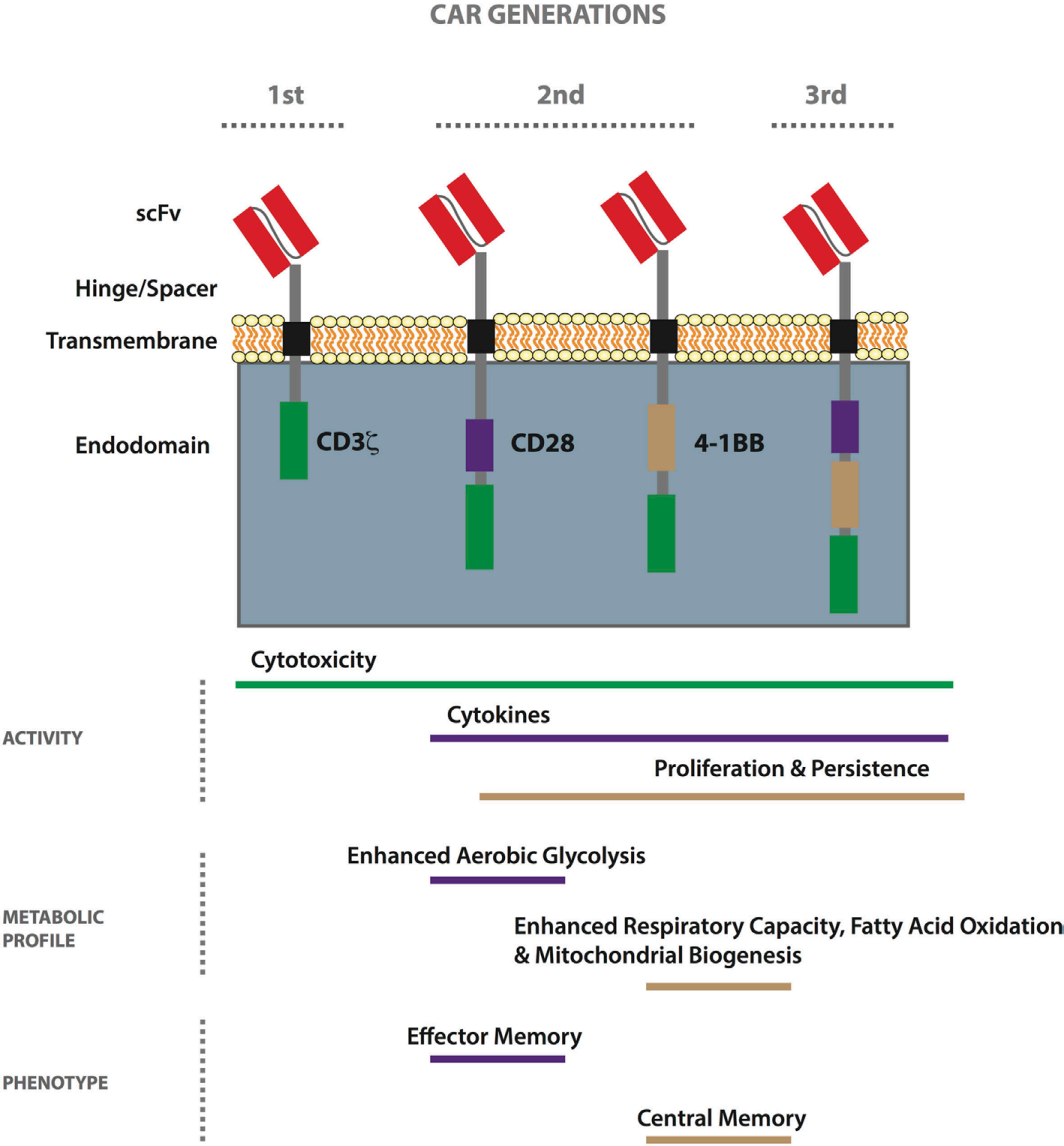
**Properties of first-, second-, and third-generation chimeric antigen receptor (CAR) T-cells**. CARs comprise an extracellular tumor-binding moiety, typically a single-chain variable fragment (scFv), followed by a hinge/spacer of varying length and flexibility, a transmembrane (TM) region, and various combinations of endodomains associated with T-cell signaling. While first-generation CARs include the endodomain of CD3ζ, second- and third-generation CARs also have one or more costimulatory endodomain, respectively. The most commonly used costimulatory endodomains are from CD28 and 4-1BB. CAR T-cell functionality can vary based on design (scFv affinity, hinge/spacer length, TM domain, etc.) and generation. In general, the 4-1BB endodomain confers the highest level of *in vivo* persistence and resistance to exhaustion. Metabolically, second-generation CD28-based CAR T-cells exhibit enhanced aerobic glycolysis as compared to 4-1BB-based ones which demonstrate enhanced respiratory capacity, fatty acid oxidation, and mitochondrial biogenesis. Stimulated CD28-based CAR T-cells acquire an effector memory-like phenotype, whereas 4-1BB-based ones take on a central memory-like phenotype.

Chimeric antigen receptors, first conceived in the late 1980s (60), are hybrid receptors comprising (i) an extracellular tumor-binding moiety, typically an Ab-derived single-chain variable fragment (scFv), (ii) a hinge/spacer, (iii) a transmembrane (TM) region, and (iv) various combinations of intracellular signaling domains associated with T-cell activation (61). First-generation CARs include the endodomain of CD3ζ only (for signal 1 of T-cell activation), while second- and third-generation CARs also have one or more costimulatory endodomains (for signal 2), respectively (Figure 1) (62). Finally, armored CAR T-cells are further gene modified to express or block molecules and/or receptors to enhance immune activity. Patient responses to first-generation CAR T-cells were disappointing, probably due to poor expansion and persistence (63–65) as a result of an anergic phenotype (66–68), and most ongoing trials involve second-generation CARs incorporating either CD28 or 4-1BB (CD137) (39, 69). CARs can be transiently expressed in primary T-cells by RNA electroporation, typically for about 1 week with current technology, or they can be stably incorporated into the genome by lentiviral or gamma-retroviral transduction (70), as well as by transposon/transposase-mediated integration using the sleeping beauty system (71). RNA electroporation along with dosing escalation is often used in the testing of new CARs in the clinic. To minimize toxicity, molecules secreted by armored CAR T-cells can be placed under an inducible promoter (72). To date, there have been no safety issues related to viral or transposon-mediated genomic integration of CARs (38, 73).

Unlike T-cell receptors (TCRs) that are MHC restricted, CARs can potentially bind any cell surface-expressed antigen and can thus be more universally used to treat patients. CARs have been developed against not only proteins, including the pMHC complex (74), but also against carbohydrates and glycolipids, as well as targets upregulated in the tumor stroma (75) and the tumor vasculature (42). Notably, not all scFvs are suitable for CAR development. A recent study demonstrated antigen-independent clustering of an anti-GD2 CAR, caused by the framework region sequences, thereby exhausting the cells and limiting antitumor responses. Interestingly, it was further shown that 4-1BB was superior to CD28 in alleviating exhaustion from this tonic signaling (76). In addition, caution should be taken in the use of scFvs of murine origin, as human-anti-mouse Ab responses can deplete the transferred CAR T-cells, and even result in patient death by anaphylactic shock following multiple infusions (77, 78). Interestingly, similar to TCRs, increasing receptor-binding strength can augment T-cell function, but there is an affinity threshold beyond which there is no further gain in activity, and target density impacts CAR T-cell activation (79–81). Along with scFv, other tumor-binding moieties including an anti-integrin peptide α5β6 (82), heregulin (83), interleukin 13-zetakine (84), NKG2D (85), vasculature endothelial growth factor (VEGF) (86), and TCRs (87) have been incorporated into functional CARs. Finally, universal CARs, including an avidin ectodomain (88) and an anti-FITC scFv (89) for recognizing targets bound by biotinylated and FITC-labeled mAbs, respectively, have been developed.

### CAR T-Cell Safety and Next-Generation Receptor Design

Chimeric antigen receptor T-cells are a potent living drug and a primary consideration in their development is the choice of target antigen. Ideally, it is highly expressed on the tumor and not at all on normal cells. Currently, there are about 30 solid tumor antigens being evaluated for CAR therapy including mesothelin, carcinoembryonic antigen (CEA), the diganglioside GD2, interleukin 13 receptor alpha (IL13Ra), human epidermal growth factor 2 (HER-2), fibroblast-activating protein (FAP), and L1 cell adhesion molecule (L1CAM) (90, 91). Unfortunately, there have been instances of severe on-target/off-site toxicity (92) such as a HER-2 CAR that may have caused patient death *via* reactivity with low levels of cognate antigen expressed on lung epithelium (93). One approach to circumvent this is to use a lower affinity scFv such that the CAR T-cells are only activated in the presence of high cell-surface expression of antigen (i.e., on the tumor cells only) (94–96). Or one can target an antigen that is tumor restricted, such as epidermal growth factor receptor variant III (EGFRvIII) (97), but there are few such examples.

In recent years various novel, next-generation engineering strategies have been devised to improve CAR T-cell safety. For example, the signaling can be split by coexpressing two CARs, one incorporating CD3ζ and the other the costimulatory endodomain, such that the T-cell will only be fully activated when both receptors are engaged (98, 99). Bispecific tandem CARs (TanCARs; the extracellular domain of one receptor can engage two distinct antigens) (100) have also been developed that synergistically enhance T-cell activity levels when coengaged. More recently, novel synthetic Notch-based receptors have been designed that enable combinatorial activation of T-cells—binding by the Notch-based CAR upregulates expression of the second CAR (101, 102). In addition, innovative ON-Switch/Remote Control CARs have been developed that restrict T-cell activation to tumor cell antigen encounter in the presence of a remotely provided heterodimerizing small molecule that links the antigen-binding receptor with intracellular components that initiate signaling (103). Finally, it is also possible to gene-modify CAR T-cells with various suicide genes or safety switches such as inducible caspase 9 to cause rapid T-cell destruction in the case of an adverse reaction in a treated patient (104, 105). In the event of CAR T-cell toxicity, such as cytokine release syndrome (CRS), early and aggressive supportive patient care is critical. The current mainstay treatment for CRS is IL-6 receptor blockade with the monoclonal Ab tocilizumab, but in the case of neurologic toxicities corticosteroids are employed (106).

### CAR T-Cell Activity As a Function of Receptor Design

The distance between a T-cell and its target antigen-presenting cell (APC) of approximately 15 nm is dictated by the TCR–pMHC interaction and is critical for the exclusion of large phosphatases such as CD45 and CD148 to the periphery of the immune synapse (107), thereby enabling TCR clustering and triggering (1). The precise mechanism by which CARs activate T-cells has not been fully elucidated, but the hinge/spacer, which governs the spatial distance between a CAR T-cell and its target, can significantly impact function (108, 109). Various hinge/spacers have been incorporated into CARs including regions from CD8α and CD4, as well as CH2–CH3 from the Fc domains of IgG1 and IgG4. In the case of CH2 spacers derived from IgG4, sequence modification is required to prevent Fc receptor binding by myeloid cells that can cause activation-induced T-cell death (AICD) (110, 111). Most CARs comprise a TM domain from a type I membrane protein such as CD4, CD8, CD3ζ, or CD28. Interestingly, it was demonstrated that CARs comprising a CD3ζ TM domain engage endogenous TCR–CD3 complexes for optimal activity (112). CARs can function, however, in the absence of endogenous TCR (113), and important efforts are being undertaken to develop universal allogeneic T-cell donors for immunotherapy (114).

In general, distal membrane epitopes require a shorter hinge/spacer, whereas an epitope that is closer to the tumor cell membrane may necessitate a longer and more flexible one to facilitate scFv binding and optimal T-cell activity (115). Recently, a Strep-tag II has been used to modify spacer length and at the same time provide a means of identifying and rapidly purifying CAR-engineered T-cells, during both the manufacturing process and patient monitoring (116). A notable study comparing three L1CAM CARs varying only in hinge/spacer length (short, medium, and long) revealed that the CAR conferring the highest *in vitro* function (long; IgG4 hinge–CH2–CH3 spacer) fared poorly *in vivo*, and, conversely, the weakest CAR *in vitro* (short; IgG4 hinge) performed the best in tumor-bearing mice. The authors hypothesized that the limited duration of CAR signaling required for *in vitro* assays does not reflect the recursive rounds of activation needed to eradicate a tumor *in vivo*. Thus, they set up a coculture stress test for which CAR T-cells were repeatedly harvested and transferred to culture dishes seeded with fresh tumor cells. Interestingly, after three rounds, the CAR T-cells bearing the long linker (i.e., conferring the best activity in round 1) underwent the highest level of AICD as a result of upregulated FasL–Fas interactions (110). This work highlights a disaccord between T-cell responses observed in standard *in vitro* testing versus *in vivo* challenges. Establishing *in vitro* T-cell parameters such as receptor affinity/kinetics and two-dimensional interactions (117), functionality upon stress testing (110), gene expression profiles upon activation, etc., that correlate to maximum antitumor responses *in vivo* is an important area of research for the efficient screening of new leads for preclinical and clinical testing.

### Optimal T-Cell Subsets for CAR Activity

**Figure 2:**
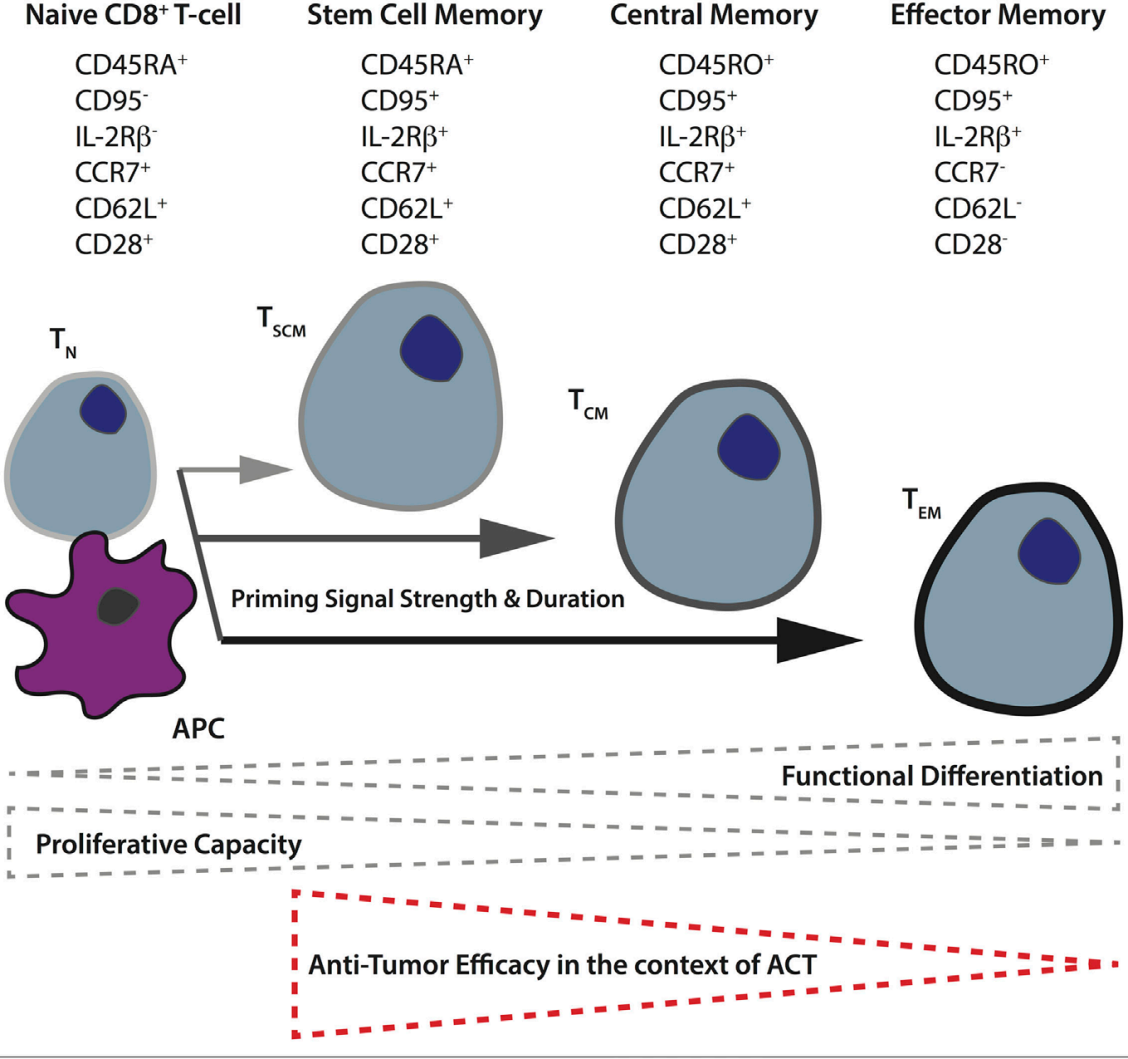
**Differentiation of naïve T-cells to memory subsets upon priming in secondary lymphoid organs**. The differentiation state of T-cells depends upon the strength and duration of priming naïve T-cells receive from antigen-presenting cells in the secondary lymphoid organs. In general, the more differentiated the T-cell, as a result of more robust priming, the lower its proliferative and self-renewal capacity, and the poorer its antitumor control in the context of adoptive T-cell therapy. The different memory T-cell subsets can be distinguished based on their expression of various cell-surface markers including the tyrosine phosphatase CD45 (full length = CD45RA, truncated form = CD45RO), the lymph node homing receptors, chemokine receptor 7, and CD62 ligand, as well as CD95 (Fas receptor), interleukin-2 receptor β-chain, and the costimulatory receptor CD28.

Another important factor governing CAR T-cell activity *in vivo* is the subset of input T-cells used. Based on their differentiation and level of maturity, T-cells are presently classified into naïve (T_N_), and four main activated subtypes: stem cell memory (T_SCM_), central memory (T_CM_), effector memory (T_EM_), and terminally differentiated effector cells (T_EFF_) (118). For both mouse and humans, it has been demonstrated that the less differentiated subsets (T_SCM_ and T_CM_) display better expansion, persistence, and antitumor activity *in vivo* (119–122). The phenotypic and functional properties of human CD8^+^ T_N_ versus the different memory subsets are illustrated in Figure 2. Retrospective studies from ACT trials have correlated objective clinical responses in patients with the transfer of less differentiated T-cells (123, 124). A recent analysis of CD19 CAR-treated patients revealed a correlation between *in vivo* expansion and the level of infused T_SCM_ phenotype (125). There are also studies demonstrating better antitumor responses when both CD4^+^ and CD8^+^ CAR T-cells are transferred (126), and it has proven beneficial to gene-engineer virus-specific memory T-cells that can persist long term, presumably *via* TCR-mediated survival signals and the quality of the original TCR-mediated priming of the cell (127). Although T-cell metabolism has long been an active field of study due to the remarkable cellular rewiring required to accommodate changes in energetic requirements when naïve, quiescent T-cells encounter and respond to cognate pMHC, it is only recently that it has come to the forefront of cancer research.

## Interplay Between the Metabolic Activity of T-Cells and Function

### Metabolic Activity of Naïve T-Cells

**Figure 3:**
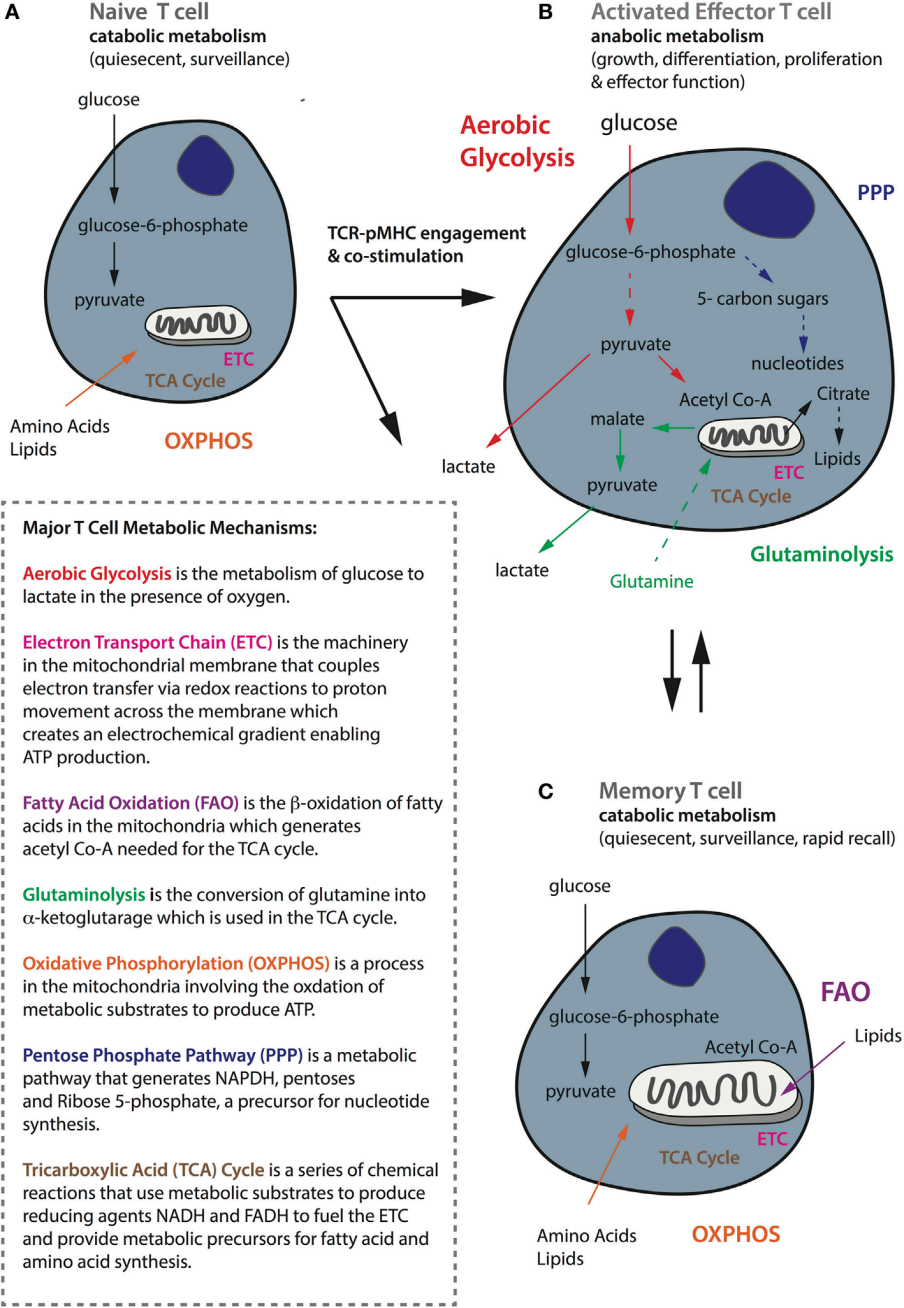
**General metabolic profiles of naïve, activated, and memory T-cells**. **(A)** Naïve T-cells are quiescent and perform catabolic metabolism to accommodate basic energy requirements. **(B)** Activated effector T-cells, whereas switch to anabolic metabolism to meet increasing energy demands as well as for biosynthesis to support functionality including proliferation, growth, and cytokine secretion. **(C)** Memory cells, like naïve cells, are quiescent and perform metabolic catabolism. While naïve and memory T-cells mostly rely on oxidative phosphorylation (shown in orange), activated ones are metabolically rewired to upregulate aerobic glycolysis (shown in red). Activated T-cells also require glutaminolysis (in green) and the pentose phosphate pathway (in navy blue) to support their activities. Memory T-cells rely on fatty acid oxidation (in purple) and they have increased mitochondrial mass and maintain substantial spare respiratory capacity to enable survival and rapid recall to antigen challenge. A brief summary of the major metabolic mechanisms is described in the inlet.

All T-cells use glucose as their primary source of fuel for the generation of adenosine triphosphate (ATP), but there are important differences in the metabolic requirements and pathways used by naïve, activated, and memory T-cells, which correspond to their specific functional state (128). T_N_ cells are quiescent and have limited biosynthetic needs because their major role is to circulate the host with the aim of being primed by an APC. Quiescent T-cells, comprising both naïve and memory T-cells, rely on catabolic metabolism, whereby nutrients including glucose, fatty acids, and amino acids are broken down for fuel (128). Indeed, quiescent T-cells mainly undertake oxidative phosphorylation (OXPHOS), a process that takes place in the mitochondria involving the oxidation of substrates in the tricarboxylic acid (TCA) cycle to generate ATP (129) (summarized in Figure 3A). The TCA cycle itself is a series of chemical reactions that use metabolic substrates to produce reducing agents such as the coenzyme reduced nicotinamide adenine dinucleotide (NADH), which donate electrons to the electron transport chain. Up to 36 molecules of ATP can be generated by OXPHOS per glucose molecule.

### Metabolic Activity of Effector T-Cells

Upon productive TCR–pMHC engagement and costimulation, naïve T-cells become activated and undergo extensive metabolic rewiring causing them to enlarge in size, and enabling extensive proliferation and the acquisition of effector functions such as cytokine secretion (130). Activated T-cells switch to *anabolic* metabolism meaning that nutrients are used to construct molecular building blocks. This transition is associated with mTOR induction, and the expression of the transcription factors Myc and hypoxia-inducible factor-1α (HIF-1α) (131, 132). Although activated T-cells increase mitochondrial OXPHOS and reactive oxygen species production, they also upregulate and rely heavily upon aerobic glycolysis (133) to meet their metabolic needs (134, 135) (Figure 3B). Upon T-cell activation, there is an increase in the expression of the glucose receptor Glut1, as well as glycolytic enzymes to enable increased import of glucose that is converted to glucose-6-phosphate and eventually pyruvate that is finally secreted in the form of lactate. It has recently been demonstrated that T-cells activated under hypoxic conditions upregulate significantly higher levels of Glut1 than under atmospheric oxygen (136), but the reactivation of CD8^+^ T-cells under hypoxia has also been shown to switch them toward a poorly proliferative and IL-10 secreting phenotype (137). This phenomenon of aerobically fermenting glucose to lactate, despite sufficient oxygen to support OXPHOS, a process also utilized by tumor cells, which themselves are heavy consumers of glucose, is known as the Warburg effect (138, 139).

Aerobic glycolysis is energetically less efficient than OXPHOS, generating only two molecules of ATP per glucose molecule, but the process is rapid and in addition supplies a critical source of metabolic intermediates needed for the synthesis of nucleic acids, proteins, carbohydrates, and lipids, and it provides a means of maintaining the NAD^+^-NADH redox balance (138). Glycolytic metabolites also help to sustain effector functions through transcriptional and translation regulation (59). For example, glyceraldehyde-3-phosphate promotes IFNγ production by relieving its restraint by glyceraldehyde-3-phosphate dehydrogenase (140). In addition, Ho et al. demonstrated that phosphophenolpyruvate (141) accumulation can help sustain the Ca^2+^-NFAT pathway, which controls the production of effector molecules, by inhibiting ER Ca^2+^ reuptake. It has been shown that activated T-cells can switch between OXPHOS and aerobic glycolysis depending on their environment, but glycolysis is required for full effector function—if activated T-cells revert to OXPHOS it will be at the expense of IL-2 and IFNγ production (140). In addition to glucose, activated T-cells rely upon an extracellular supply of glutamine to replenish intermediates for the TCA cycle through the process of glutaminolysis, and this can also contribute to the citrate pool used for lipid synthesis by reductive carboxylation (142, 143). Finally, activated T-cells decrease fatty acid oxidation (FAO), decrease pyruvate flux into the TCA cycle, and increase glucose flux into the pentose phosphate pathway to produce nucleotides for DNA synthesis (Figure 3B).

### Metabolic Activity of Memory T-Cells

By contrast, memory T-cells transition from aerobic glycolysis to OXPHOS, fueled in part by the catabolism of intracellular fatty acids in the mitochondria (144–146). Furthermore, memory T-cells have increased mitochondrial mass and activity (147, 148) and upregulate mitochondrial biogenesis to build substantial spare respiratory capacity (SRC), thereby enabling both survival and rapid recall to antigen challenge (130) (Figure 3C). Interestingly, memory T-cells activated *in vitro* increase aerobic glycolysis, and consequently IFNγ production, more rapidly than their naive counterparts. How this relates to their capacity to compete for glucose in the TME remains to be established (149). Moreover, memory T-cells can potentially better adapt to a nutrient crisis because they have healthier mitochondria (147). AMP-activated protein kinase (AMPK) is another important metabolic regulator in T-cells that senses a high AMP to ATP ratio and can promote catabolic pathways and conservation of energy during metabolic stress. AMPK is important for memory T-cell development (150); treating activated CD8^+^ T-cells with either rapamycin (inhibits mTOR) or metformin (activates AMPK) to augment catabolic pathways enhances CD8^+^ memory formation (151, 152).

### Immunometabolism and T-Cell Therapy

It has been observed that several genes involved in metabolism are downregulated in exhausted T-cells (153). Moreover, blocking leucine or glucose metabolism during T-cell activation leads to an anergic phenotype (154). Mechanistically, leucine can stimulate mTOR *via* leucyl-tRNA synthetase and hence low leucine levels may impair mTOR activation (155, 156). It has also been demonstrated that both PD-1 and CTLA-4 ligation inhibits glycolysis in activated T-cells but through distinct mechanisms; CTLA-4 inhibits Akt *via* the serine–threonine phosphatase PP2A, while PD-1 inhibits Akt phosphorylation by blocking CD28-mediated activation of PI3K (157). PD-1 signaling also blocks amino acid metabolism but promotes fatty acid β-oxidation (158). With an increased appreciation for the high competition for nutrients including glucose in the TME which can promote tumor progression, as well as the dynamic interplay between T-cell phenotype, metabolism, and immune checkpoint, it is clear that an important consideration in the development of CAR T-cells is how their culture prior to transfer, as well as their design, affects their metabolic profile.

Traditionally, T-cells for ACT are cultured with high doses of IL-2, followed by a rapid expansion protocol comprising agonistic anti-CD3 Ab and allogeneic feeder cells (159). However, it is now known that TCR signaling coupled with high-dose IL-2 drives T_EFF_ differentiation (160) which is not an ideal phenotype for ACT. Alternative common gamma chain (γc) signaling cytokines have also been assessed for T-cell culture. Naïve mouse T-cells cultured in the presence of IL-15 (145) or IL-21 (161), for example, acquire phenotypic, functional, and metabolic properties of naturally occurring T_CM_ cells. Moreover, IL-15 cultured murine T-cells confer superior *in vivo* antitumor activity than ones cultured in IL-2 (162). Similarly, human T-cells cultured with artificial APCs and IL-15 exhibit a T_CM_ phenotype, demonstrate clonotypic persistence, and can mediate objective clinical responses upon transfer (163, 164). Human T-cells cultured in IL-21 also maintain a minimally differentiated profile (161, 165). Interestingly, it has been demonstrated that IL-15 regulates SRC and oxidative metabolism by mitochondrial biogenesis as well as the expression of carnitine palmitoyl transferase, a metabolic enzyme involved in the rate-limiting step in FAO (145). IL-7 also enhances T-cell survival in a metabolically driven manner by Glut1 trafficking *via* STAT5 and Akt (166), as well as by inducing glycerol transport (*via* AQP9) and triglyceride synthesis (167). Finally, metabolically robust T-cells isolated with the lipophilic cationic dye tetramethylrhodamine methyl ester (TMRM; staining can be used to distinguish mitochondrial membrane potential) conferred enhanced persistence and tumor eradication upon ACT (168).

A T-cell’s commitment between a memory versus effector phenotype is governed by its metabolic state (145, 152, 169), which in turn is controlled by stimuli from TCR, costimulatory and cytokine receptors that converge at common development, and differentiation signal transduction pathways including PI3K/Akt/mTOR and Wnt/β-catenin (130). Thus, small molecule modulators of these pathways have been assessed for the *in vitro* culture of T-cells for ACT. Promotion of the canonical Wnt/β-catenin pathway with the GSK3β inhibitor TWS119, for example, favors the formation of T_CM_ and T_SCM_ cells with improved *in vivo* antitumor responses as compared to untreated cells (170). Similarly, Akt inhibition during *ex vivo* priming and expansion of T-cells favors T_SCM_-like cells having higher rates of OXPHOS and FAO and enabling enhanced *in vivo* tumor control (171). Another consideration in T-cell culture for ACT is glucose concentration in the media. Although media often comprise about 5.5 mM glucose (similar to that of blood), many used for ACT are in the range of 10–25 mM—this may program high dependency on glucose and thereby further impair T-cell responses in the nutrient-deprived TME (169, 172). Interestingly, it has been shown that the activation of CD8^+^ T-cells in the presence of 2-deoxyglucose, an inhibitor of glycolysis, enhances memory generation and antitumor responses (169). Several amino acids have been implicated in immunomodulation including cysteine, glutamine, phenylalanine, tryptophan, and arginine (173). It has been observed that low arginine, for example, induces the loss of CD3ζ and inhibits proliferation and cytokine production by T-cells, but that this state can be reversed by exposure to excess arginine (174). Recently, it has been further demonstrated that the culture of murine T-cells with elevated l-arginine promotes the generation of T_CM_-like cells, shifts the metabolic profile from glycolysis to OXPHOS, and endows the T-cells with enhanced antitumor activity *in vivo* (175).

### CAR Endodomains and Metabolism

As previously mentioned, in recent years, there have been important clinical responses to CD19 CAR T-cell therapy against hematological tumors including ALL, chronic lymphocytic leukemia (CLL), and diffuse large B cell lymphoma, using second-generation receptors comprising either CD28 or 4-1BB [reviewed in Ref. (44)]. It is oftentimes difficult to compare the success of trials because of differences in the study design, including the scFv used, the gene transfer protocols, and interventions undertaken post-transfer. Initial clinical response rates against ALL have been the same for CD19 CAR trials incorporating either CD28 or 4-1BB (45, 176, 177). In the case of CLL, however, 4-1BB-based CAR T-cells appear superior (178), demonstrating persistence of greater than 4 years in some patients versus 30 days in the case of CD28 (179). Signaling pathways induced by CD28 versus 4-1BB, a member of the tumor-necrosis receptor family, are distinct. While CD28 activates the PI3K/Akt pathway that can enhance glycolysis (180), 4-1BB has been linked to long-term survival of T-cells (181). Recently, the metabolic pathways induced by the CD28 versus the 4-1BB costimulatory endodomain of second-generation CD19 CAR T-cells were described for the first time (182). T-cells engineered with a 4-1BB-bearing CAR had an increased frequency of T_CM_ phenotype, mitochondrial biogenesis, and oxidative metabolism upon activation, and had greater survival, as compared to the CD28-based CAR T-cells that had enhanced aerobic glycolysis and a predominantly T_EM_ phenotype (Figure 1). This work has important implications in the choice of CAR costimulatory endodomains for targeting different solid TMEs. For example, in the case of a tumor such as BRAF V600E melanoma, which has a high glycolytic rate (183), it may be prudent to develop a CAR incorporating 4-1BB. Pretreatment with a BRAF inhibitor may also help in restoring glucose levels prior to ACT for improved T-cell activity in the TME. Alternatively, it may be beneficial to transfer both CD28 and 4-1BB-based CAR T-cells for both immediate effector and persistent antitumor activity.

## Armored CAR T-Cells and Combination Therapies to Overcome Immunometabolic Obstacles in Solid Tumors

### Immunometabolic Obstacles in Solid Tumors

**Figure 4:**
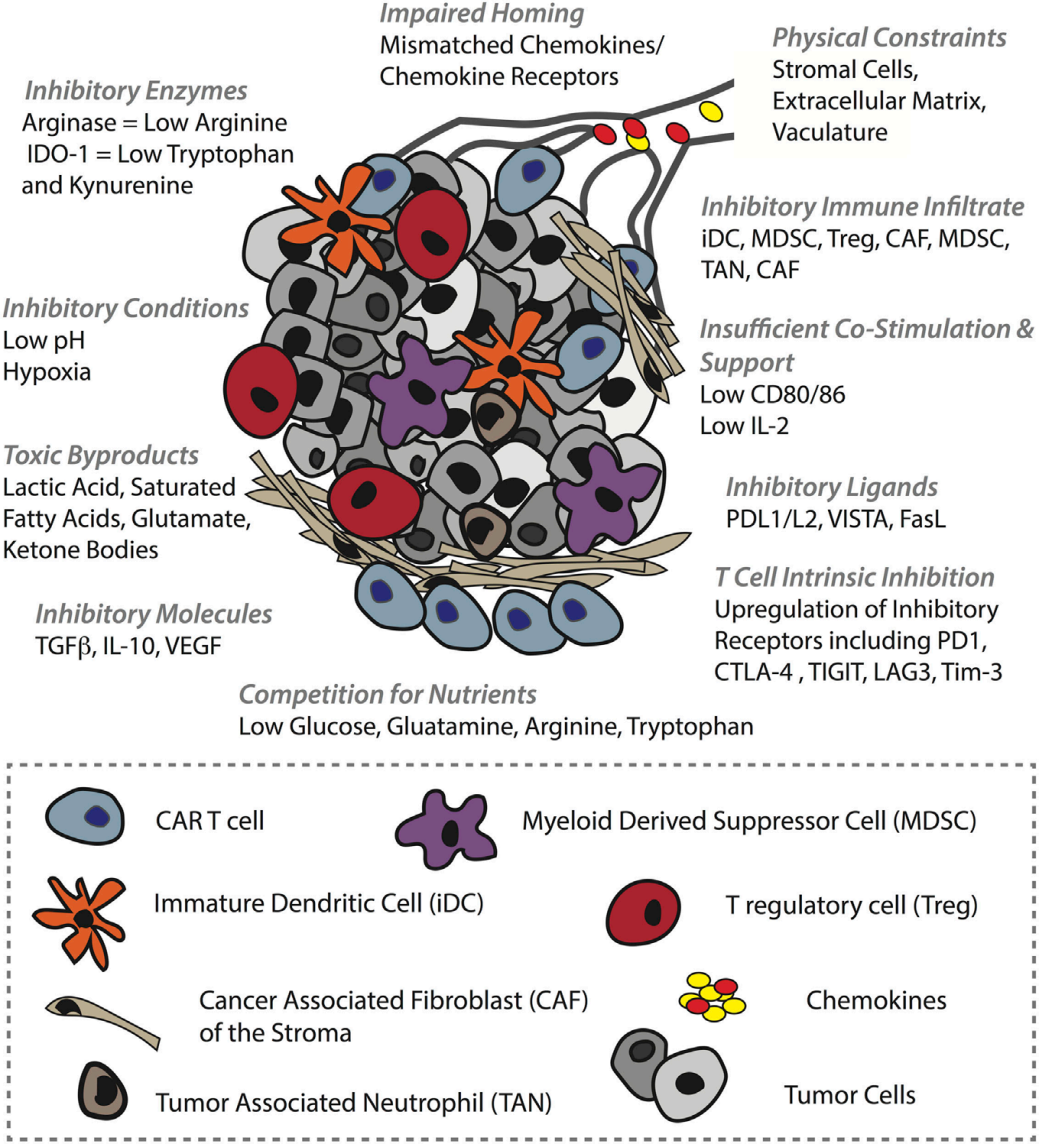
**Barriers in the solid tumor microenvironment (TME) that can hinder chimeric antigen receptor (CAR) T-cell activity**. In order to mediate an antitumor response, CAR T-cells must first successfully home to and penetrate the tumor bed. Obstacles to these events include tumor chemokine and T-cell chemokine receptor mismatches, and various inhibitory mechanisms in the vasculature such as ICAM-1 downregulation. In the stroma, T-cells can be physically blocked by dense extracellular matrix and by inhibitory cancer-associated fibroblasts. The TME itself is also hostile, comprising a range of inhibitory immune cells, inhibitory receptors such as PD-L1 on tumor cells and immune infiltrate alike, and soluble molecules such as TGFβ that impair T-cell activity (some examples are shown). In addition, the TME is typically acidic, hypoxic, full of toxic metabolites, and nutrient depleted which can inhibit not only T-cell activity but also other immune infiltrate such as DCs that lose their ability to mature and provide support to T-cells. Moreover, some of these conditions promote protumoral immune cells.

The clinical efficacy of CAR T-cells against solid tumors remains to be proven (184). The two most successful trials to date involve HER-2 CAR T-cells against sarcoma, for which 4/17 patients showed stable disease (185), and GD2 CAR T-cells to treat neuroblastoma, for which 3/11 patients underwent a complete response (186). There are various physical and physiological hurdles faced by T-cells in the context of solid tumors (46). To begin with, T-cells must successfully, (i) home to the tumor bed, often in the face of mismatches between T-cell chemokine receptors and chemokines present in the TME. Furthermore, T-cells must migrate along, (ii) an aberrant vasculature that is not conducive to transendothelial migration of T-cells due to downregulation of adhesion molecules (ICAM-1) (42) and the upregulation of FasL (187), etc., and they can encounter, (iii) additional barriers in the stroma, including a dense collagen matrix and suppressive cancer-associated fibroblasts (47). If T-cells are successful in penetrating the tumor bed, there they can face a battery of obstacles, including (iv) suppressive immune infiltrate comprising T regulatory cells (Tregs), myeloid-derived suppressor cells (MDSCs), tumor-associated macrophages, tumor-associated neutrophils (188), and immature DCs (189), (v) a range of suppressive molecules such as transforming growth factor beta (TGFβ) VEGF and adenosine, (vi) suppressive ligands including PD-L1/L2, VISTA, and FasL (190), (vii) competition for, and downregulation of, costimulatory ligands such as CD80/86, and (viii) T-cell-intrinsic regulatory mechanisms including PD-1 and CTLA-4 upregulation, and ultimately exhaustion (191) or anergy (192). Finally, (ix) the T-cells must function in an environment that is acidic, hypoxic (193), nutritionally depleted (194) and comprising toxic metabolic by-products such as lactic acid, glutamate, and ketone bodies (195) (summarized in Figure 4).

### CAR T-Cell Coengineering and Combinatorial Therapy for Tumor Homing and Migration into the Tumor Bed

**Figure 5:**
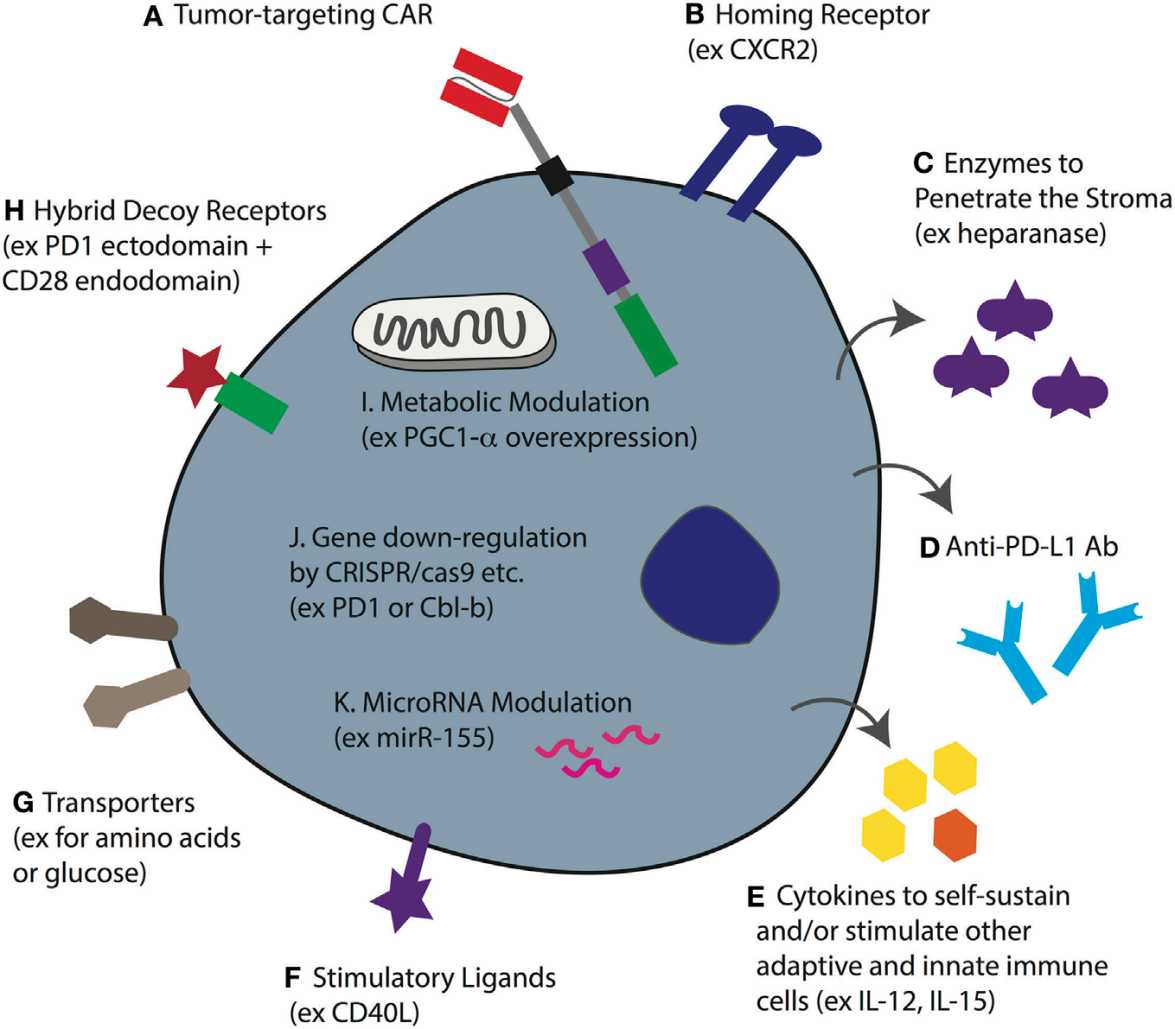
**T-cell coengineering strategies to target and attack solid tumors, and overcome immunometabolic barriers**. Human T-cells, ideally minimally differentiated, can be gene-engineered *ex vivo* in various ways including transient RNA electroporation and stable genome integration by lentiviral transduction and sleeping beauty. **(A)** To enable tumor specificity, the T-cell can be engineered with a chimeric antigen receptor (CAR) directed against a surface-expressed tumor antigen (or a TCR against a target pMHC). **(B)** To enable tumor homing the T-cell can be coengineered to express a specific chemokine receptor that matches the chemokine profile of the tumor being targeted. **(C)** To better penetrate dense stroma CAR T-cells can be coengineered to secrete heparanase. **(D)** Instead of injecting the patient with checkpoint blockade monoclonal antibodies (mAbs), the T-cells can be gene engineered to secrete anti-PD ligand 1 (PD-L1) mAb. **(E)** The tumor microenvironment (TME) often lacks T-cell promoting cytokines, either because they are not produced, or because they are competitively consumed, such as IL-2 by T regulatory cells. Thus, CAR T-cells can be coengineered to forcibly secrete various cytokines that can serve not only to self-sustain the CAR T-cell but also to stimulate other adaptive and innate immune cells in the TME. **(F)** Forced expression of the stimulatory ligand CD40L will help activate antigen-presenting cells as well as upregulate adhesion molecules on endothelial cells. **(G)** The upregulation of nutrient transporters for glucose or amino acids may help to increase T-cell competition for limited resources in the TME. **(H)** Hybrid receptors such as ones comprising the extracellular domain of PD-1 fused to the endodomain of CD28 can turn a negative signal (i.e., from PD-L1) into one that costimulates the cell. This may be further improved by designing a higher affinity variant of PD-1. **(I)** The overexpression of intracellular proteins such as the transcription coactivator PGC1-α, can help to reverse metabolic exhaustion. **(J)** Using various techniques including CRISPR/cas9, it is possible to knockout gene expression such as of PD-1 and the master regulator Cbl-b to enhance T-cell activity in the TME. **(K)** microRNAs play an important role in T-cell activity and the overexpression of miR-155, for example, can be used to enhance sensitivity to homeostatic γc cytokines. Note that some of these approaches have yet to be demonstrated for engineered CAR T-cells.

The predominant chemokine receptor mediating effector T-cell recruitment to tumors is CXCR3 *via* chemokines CXCL9 and CXCL10 (196). Chemokines commonly secreted in TMEs, however, rather than attracting cytotoxic T-cells often recruit inhibitory immune cells such as Tregs and MDSCs, whose presence is associated with poor patient prognosis (197). The chemokine CCL22, for example, present in breast and prostate cancer, mediates CCR4-dependent Treg trafficking (197, 198), while hypoxia-dependent expression of the chemokine ligand CCL28 in ovarian cancer recruits Tregs *via* CCR10 (199), and in pancreatic cancer the upregulation of CCL5 induces the migration of CCR5-expressing Tregs (200). Tregs are potent inhibitors of CD8^+^ T-cells as they compete for IL-2 (201), generate adenosine by CD39/CD73 (202), and *via* CTLA-4 downregulate CD80/CD86 on DCs that are needed by T-cells for costimulation (203) while upregulating indoleamine 2,3-dioxygenase-1 (IDO-1) in DCs (204, 205). MDSCs can be recruited to different TMEs by CCL2, CXCL5, CXCL12, and stem cell factor (206). They also inhibit tumor immunity by various mechanisms including the expression of arginase, TGFβ, cyclooxygenase 2 that controls prostaglandin E_2_ (PGE_2_) production (207) (a powerful repressor of TCR signaling), and IL-10. In addition, MDSCs can sequester cysteine and induce Tregs (188). One approach to direct CAR T-cells (Figure 5A) toward a tumor that does not express CXCL9 or CXCL10 is to coengineer them with a borrowed chemokine receptor (Figure 5B). For example, CXCR2-engineered T-cells (208, 209) demonstrated improved localization and control of melanoma tumors expressing CXCL1 and CXCL8, chemokines that enable the migration of CXCR2^+^ monocytes.

In order to tackle the stroma, FAP-directed CAR T-cells have been developed, and in murine tumor models have been shown to slow tumor growth (49). The observation that *in vitro* cultured T-cells downregulate heparanase, an enzyme that is required for the degradation of heparin sulfate proteoglycans, the primary component of the extracellular matrix of tumor stroma, led to the development of CAR T-cells coengineered to secrete it (210) (Figure 5C). Such CAR T-cells may be potent against stroma-rich solid tumors. Various approaches can be taken to normalize the tumor vasculature (42). For example, blocking endothelin B receptor (211), and the pharmacologic inhibition of VEGF and PGE_2_ to attenuate FasL expression, enables enhanced CD8^+^ T-cell influx and tumor control (187). In addition, CAR T-cells that disrupt the tumor vasculature and mediate tumor regression have been developed (212).

### CAR T-Cell Coengineering and Combinatorial Therapy for Overcoming Immunometabolic Challenges in the Tumor Bed

The solid TME is hostile for effector T-cells. As a result of high aerobic glycolysis by tumor cells, as well as the fact that nutrients and oxygen must be supplied, and waste removed, by an aberrant vasculature, tumors are typically nutrient depleted, hypoxic, acidic, and toxic. As previously described, low glucose levels and a lack of critical amino acids such as leucine and arginine will alter T-cell metabolism and directly impair their function (156, 213, 214). Gene-engineering approaches to render CAR T-cells more competitive in nutrient acquisition, such as by overexpressing transporters (Figure 5G), or to rewire their metabolism, may improve their activity in solid tumors. For example, overexpression of PPAR-gamma coactivator 1-α (PGC1-α), a transcriptional coactivator involved in mitochondrial biogenesis, could in part reverse metabolic exhaustion (decreased mitochondrial mass and function induced by chronic Akt signaling) of T-cells in the TME (215) (Figure 5I). Interestingly, modulating cholesterol metabolism can enhance antitumor response of CD8^+^ T-cells; both pharmacological inhibition and gene knockdown of the cholesterol esterification enzyme ACAT1 increased plasma membrane concentration of cholesterol, enabling more efficient immune synapse formation, TCR clustering, and enhanced signaling (216). Whether such strategies would also augment CAR T-cell activity remains to be determined.

Various other immunometabolic gene-engineering strategies have been proposed for increasing the activity of TCR- or CAR-engineered T-cells in the TME. With respect to the PD-1/PD-L1 checkpoint blockade axis, at least three different approaches have been taken. PD-1 has been knocked down in T-cells (217, 218), hybrid receptors comprising the ectodomain of PD-1 and the endodomain of CD28 have been expressed to divert PD-L1 binding toward costimulatory intracellular signaling (219), and CAR T-cells have been engineered to secrete anti-PD-L1 Abs (220), all of which have been reported to increase antitumor responses (Figures 5D,H,J). Others have knocked-down master regulators of T-cell activity such as the E3 ubiquitin ligase Cbl-b (Figure 5J) and have shown enhanced antitumor T-cell responses (221, 222). microRNAs, such as miR155, have also been manipulated for enhanced tumor control (223, 224) (Figure 5K), and T-cells have been gene engineered to overexpress cytokines including IL-12 (225) and IL-15 (226) (Figure 5E) for improved activity. The advantage of secreted molecules is that they can support not only the T-cell that produces them but also endogenous immune cells in the TME. As a final example, coengineering CAR T-cells to constitutively express CD40L demonstrated enhanced T-cell proliferation and secretion of pro-inflammatory cytokines (Figure 5F). The CD40L^+^ CAR T-cells also increased the immunogenicity of CD40^+^ tumor cells through the upregulation of costimulatory, adhesion, and human leukocyte antigen molecules, as well as the Fas death receptor, and they induced the maturation and secretion of IL-12 by monocyte-derived DCs (58).

The TME can also reprogram other immune infiltrate to the detriment of T-cell activity. For example, the maturation, function, and phenotype of DCs can be impaired by VEGF (227), IL-6, macrophage colony-stimulating factor (228), and TGFβ (229). In addition, PD-L1 expression by DCs can be induced by IL-10 and VEGF (230). It was also recently demonstrated that HIFα expression [the main transcriptional factors responding to limited oxygen supply (231)] elevates miR-210 in MDSCs, which in turn increases both arginase activity and the production of nitric oxide (232). Along with hypoxia (233), lactate, a major byproduct of aerobic glycolysis in tumor cells, can also directly and indirectly inhibit cytotoxic T lymphocyte activity. Indeed, high lactate in the TME can block the export of endogenous lactate produced by aerobic glycolysis in T-cells *via* the gradient-dependent transporter monocarboxylate transporter-1, and thereby disturb T-cell metabolism (234). Lactic acid can also promote M2-polarization and expression of arginase-1 by HIF-1α stabilization (235). Moreover, hypoxia, *via* HIF-1, can induce glycolysis as well as a switch from glucose to glutamine as the major substrate for FA synthesis in tumor cells (236), further depleting the TME of vital nutrients needed for T-cell function. Thus, pretreatment of tumors with inhibitors of either HIF-1 or metabolic enzymes could potentially impair the metabolic flexibility of cancer cells and inhibitory immune infiltrate, thus rendering tumors more sensitive to CAR T-cell transfer—the CAR T-cells will benefit from entering a more nutrient replete and less aggressive/suppressive TME. Alternatively, metabolic drugs could be targeted to tumors with Abs, or the pharmacologic inhibitors could be designed in such a way that they are preferentially taken up by tumor cells.

A range of other strategies can be used to pretreat or cotreat tumors for enhanced responses to CAR T-cell therapy. Localized RT, for example, can reprogram the TME by various mechanisms including by inducing immunogenic cell death (52, 237), supporting T-cell trafficking to the tumor (238), and by promoting the polarization of macrophages (239) from a suppressive M2 (240) to M1 phenotype (241). An inhibitor of DNA methyltransferase 1 (catalyzes the methylation of genetic loci), 5-aza-2′-deoxycytidine, has been used to enhance expression of the epigenetically silenced chemokines CXCL9 and CXCL10 in the TME and thereby promote T-cell infiltration and responses to checkpoint blockade (242). CAR T-cells expressing CXCR3 could benefit from such treatment. Cyclophosphamide, such as RT, can be used not only to direct tumor cell destruction but also to deplete Tregs from the TME and thereby enhance responses to immunotherapy (51). As a final example, IDO-1 inhibition is a powerful approach for promoting tumor immunity. IDO-1, the rate-limiting enzyme involved in the conversion of the essential amino acid tryptophan to its catabolic product kynurenine (Kyn) (204, 243), can be upregulated in DCs, by Tregs as mentioned above, as well as by tumor cells and myeloid cells in response to IFNγ (244, 245), and has been associated with poor prognosis for several types of cancer including ovarian (246), endometrial (247), colorectal (248), and lung (249). The depletion of tryptophan in the TME activates stress response kinases in T-cells, including general control non-depressing 2 (GCN2), which detects uncharged tRNAs, and ultimately blocks T-cell proliferation and triggers the caspase pathway (250, 251). In addition, Kyn binds the arylhydrocarbon receptor, a ligand-activated transcription factor that promotes the polarization of naïve T-cells toward a Treg phenotype (251). Thus, IDO-1 inhibition promotes tryptophan availability for effector T-cells and limits Tregs in the TME.

## Concluding Remarks

Metabolism is an important driver of cancer progression that must be addressed in the context of CAR T-cell immunotherapy to improve clinical responses against solid tumors. High levels of aerobic glycolysis by tumor cells leads to an accumulation of metabolic by-products that, along with oxygen deprivation and low pH, can drive protumoral activity of various immune cells, including Tregs and M2 macrophages, as well as directly inhibit effector T-cell function. In addition, competition for critical nutrients, such as the amino acids tryptophan, glutamine, and arginine, as well as glucose, all contribute to the suppression of T-cell activity. Checkpoint pathways are intimately linked with the metabolic status of both tumor cells and T-cells; non-responsiveness to anti-PD-1 mAb has been linked to the upregulation of a metabolic gene-signature in tumors (35), whereas successful PD-L1 blockade has been demonstrated to block tumor cell glycolysis, thereby enhancing T-cell fitness (34).

Immunometabolic barriers can be targeted therapeutically prior to and/or during ACT to enhance responses to CAR T-cell therapy and to support endogenous immunity. In addition, CAR T-cells can be optimally designed based on the metabolic properties of the tumor being targeted and cultured to promote a less differentiated, long-lived phenotype that can efficiently self-renew and differentiate *in vivo* into potent effector cells (252, 253). Further, CAR T-cells can be coengineered to enhance both their own activity and that of other immune cells in the TME. Emerging knowledge on the immunometabolic pathways regulating T-cell function in tumors offers new opportunities for gene-engineering to drive favorable T-cell energetics and optimize their activity. Next-generation CAR T-cell immunotherapy based on combinatorial engineering and treatments to reprogram T-cell properties and the TME offer unprecedented hope for curing solid tumors.

## Author Contributions

MI and GC conceived the manuscript; revised and approved the final manuscript. MI, RS, KS, and ND drafted the manuscript.

## Conflict of Interest Statement

The authors declare that the research was conducted in the absence of any commercial or financial relationships that could be construed as a potential conflict of interest.
